# Digital PCR analysis of circulating tumor DNA: a biomarker for chondrosarcoma diagnosis, prognostication, and residual disease detection

**DOI:** 10.1002/cam4.1146

**Published:** 2017-08-23

**Authors:** Alice Gutteridge, Victoria M. Rathbone, Rebecca Gibbons, Mark Bi, Nicholas Archard, Kate E. J. Davies, Jake Brown, Vincent Plagnol, Nischalan Pillay, Fernanda Amary, Paul O'Donnell, Manu Gupta, Roberto Tirabosco, Adrienne M. Flanagan, Tim Forshew

**Affiliations:** ^1^ University College London Cancer Institute London United Kingdom; ^2^ Royal National Orthopaedic Hospital NHS Trust (Histopathology) Stanmore Middlesex United Kingdom; ^3^ Department of Genetics Yale School of Medicine New Haven CT 0651, USA; ^4^ Howard Hughes Medical Institute Yale School of Medicine New Haven CT, USA; ^5^ Royal National Orthopaedic Hospital NHS Trust (Radiology) Stanmore United Kingdom

**Keywords:** Chondrosarcoma, circulating tumor DNA, IDH1, digital PCR

## Abstract

Conventional chondrosarcoma is the most common primary bone tumor in adults. Prognosis corresponds with tumor grade but remains variable, especially for individuals with grade (G) II disease. There are currently no biomarkers available for monitoring or prognostication of chondrosarcoma. Circulating tumor DNA (ctDNA) has recently emerged as a promising biomarker for a broad range of tumor types. To date, little has been done to study the presence of ctDNA and its potential utility in the management of sarcomas, including chondrosarcoma. In this study, we have assessed ctDNA levels in a cohort of 71 patients, 32 with sarcoma, including 29 individuals with central chondrosarcoma (CS) and 39 with locally aggressive and benign bone and soft tissue tumors, using digital PCR. In patients with CS, ctDNA was detected in pretreatment samples in 14/29 patients, which showed clear correlation with tumor grade as demonstrated by the detection of ctDNA in all patients with GIII and dedifferentiated disease (*n* = 6) and in 8/17 patients with GII disease, but never associated with GI CS. Notably detection of ctDNA preoperatively in GII disease was associated with a poor outcome. A total of 14 patients with CS had ctDNA levels assessed at multiple time points and in most patients there was a clear reduction following surgical removal. This research lays the foundation for larger studies to assess the utility of ctDNA for chondrosarcoma diagnosis, prognostication, early detection of residual disease and monitoring disease progression.

## Introduction

Conventional central chondrosarcoma (CS) is the most common bone sarcoma in adults with an incidence of ~1/100,000/year. Clinical outcome has not changed in 30 years and tumor grade (G) is the best prognosticator of metastatic disease, and survival, aside from when the lesions occur in the phalanges as these have a negligible risk of metastasis. Enchondromas and CS GI (classified as well differentiated cartilaginous tumors when grouped together) in a long bone have an excellent prognosis and can generally be cured by curettage and local adjuvant therapy. In contrast, high‐grade disease (combined GII and GIII) has a ~53% 5 years survival rate but when assessed according to grade the 5 and 10 years survival rate of GII is 81% and 64%, and GIII is 43% and 29%, respectively 1. Approximately 10% of cases that recur reveal an increase in grade. It is however well recognized that there is significant inter‐ and intra‐observer variability when grading these tumors 2. About 10–15% of CS transform into a nonconventional variant known as dedifferentiated CS associated with a low 5 years survival, ranging from 7% to 24% 3. Typically resistant to chemotherapy and radiotherapy, GII and III disease are treated by *en bloc* excision and a prosthetic implant when required. Patients with dedifferentiated CS may be treated with chemotherapy 4.

Patients with CS would benefit from being provided with a more accurate prognosis particularly for those diagnosed with GII disease. A biomarker that would detect residual disease directly postsurgery, and earlier detection of disease relapse than is achieved using current imaging technologies, would also improve the clinical management of these patients. In addition, a blood‐based biomarker that could be employed in the absence of a CT‐guided biopsy would reduce the number of invasive tests. Development of an assay to detect circulating tumor DNA (ctDNA) in patients with CS could address these challenges as it is now well established that a broad range of cancer types release mutant DNA into the blood stream 5: this is being extensively studied in many tumor types for the applications listed above 6, 7, 8, 9. To the best of our knowledge there have only been two studies that have analyzed ctDNA in sarcoma to date: both studies assessed high‐grade disease including a single patient with widespread osteosarcoma 10 and a cohort of 20 patients with Ewing sarcoma 11.

*Isocitrate dehydrogenase type 1 (IDH1)* and *IDH2* mutations are present in close to 60% of central cartilaginous tumors: the former is present in 50–55% with *IDH2* alterations being detected in ~6% of central lesions 12. The mutations are present ab initio and retained throughout disease progression including transformation into dedifferentiated chondrosarcoma 13. All reported *IDH1* mutations in CS have been identified in codon 132 and comprise five different nucleotide changes (R132H, C, G, S, L) 12. Hence, central conventional CS is well suited to ctDNA analysis. The same mutations are also considered to be the key driver in the development of Ollier disease and Maffucci syndrome, the most common form of enchondromatosis, a well characterized mosaic disorder 14, 15.

The primary aim of this pilot study is to determine if mutant *IDH1* DNA can be detected in the plasma of patients whose tumors harbor such mutations and whether such a test could potentially be useful as a prognostic and diagnostic marker in the future.

There are a number of other bone and soft tissue tumors which harbor recurrent point mutations including a *MYOD1* p.L122R mutation in a large percentage of spindle cell rhabdomyosarcoma 16, 17, an exceptionally rare, and aggressive high‐grade tumor. Other examples include *CTNNB1* in desmoid‐type fibromatosis 18, *GNAS* in fibrous dysplasia 19 and intramuscular myxoma 20, and *H3F3* alterations in giant cell tumor (GCT) of bone and chondroblastoma 21, 22. However, in contrast to CS these do not represent high‐grade disease. Nevertheless, it would be of interest to establish if these alterations could be detected in the plasma of affected individuals.

## Materials and Methods

### Patient recruitment and sample collection

Whole blood, blood plasma, and tissue were banked where feasible from patients attending the London Sarcoma Service with peripherally sited sarcomas, including CS. Samples from 29 patients with *IDH1‐*mutant CS were identified for this study (Table S1). Three patients had extensive Ollier disease. Pretreatment plasma was also tested from patients with spindle cell rhabdomyosarcoma (*n* = 3), giant cell tumor of bone (*n* = 24), desmoid‐type fibromatosis (*n* = 5), fibrous dysplasia (*n* = 4) and intramuscular myxoma (*n* = 6) (Table S2). Blood plasma from 12 additional individuals with lipoma was used as normal controls. Samples were obtained from the Stanmore Musculoskeletal Biobank, a satellite of the UCL/UCLH Biobank (HTA License number 12055), which was approved by the National Research Ethics Committee (Reference 15/YH/0311). This specific study was approved by the NREC‐approved UCL/UCLH Biobank Ethical Review Committee (Reference EC17.14).

The tissue diagnoses and grading of tumors were reviewed and confirmed independently by three pathologists (AMF, RT, FA) blinded to the original diagnostic report. Diagnostic criteria were based on the WHO classification 3. Tumor DNA samples from patients with CS whose plasma was analyzed were confirmed to have one of the *IDH1* R132 mutations of interest using a combination of a range of techniques including capillary sequencing, Sequenom^™^ MassARRAY and digital PCR 12, 14. Chondrosarcoma samples with *IDH2* mutations were not included in the study.

In tumor types other than chondrosarcoma, the following hotspot mutations were confirmed in the tumor samples before assessing the ctDNA: *MYOD1* mutation (p.L122R) in spindle cell rhabdomyosarcoma, *CTNNB1* mutations (p.T41A and p.S45F) in desmoid‐type fibromatosis 18, *GNAS* mutations *(p.R201H, p.R201C)* in fibrous dysplasia 19 and intramuscular myxoma 20, and *H3F3A* alterations (p.G34W) in giant cell tumor of bone 21, 22. Various methods were used to determine the mutation status of these samples, including capillary, whole genome, exome sequencing, amplicon based targeted next‐generation sequencing (ion torrent), Sequenom^™^ MassARRAY, digital PCR, and mutation‐specific restriction digestion.

Blood plasma was collected following a standard operating procedure optimized for ctDNA analysis. In brief, blood was drawn into 10 mL EDTA tubes and processed no more than 4 h after collection by a 10 min centrifugation at 4°C at 272x g, followed by centrifugation of the upper plasma layer at 20,000x g for 10 min to remove any cellular debris. Plasma was stored at −80°C until DNA was extracted.

### DNA extraction from blood plasma

DNA from plasma samples was extracted using the QIAamp^®^ Circulating Nucleic Acid kit (Qiagen, Hilden, Germany) according to the manufacturer's instructions, using an elution volume of 50 *μ*L. Eluent was passed twice through the extraction column to increase extraction efficiency. DNA was stored at −20°C until required for digital PCR analysis.

### *IDH1* multiplex assay development

Initial assay design was carried out using Beacon Designer software (PREMIER Biosoft, CA). Probes were developed targeting wild type and all common *IDH1* mutations (R132C, G, H, L and S) in central CS 12. The ability to detect all five mutations was demonstrated through analysis of tumor DNA with known mutations as assessed by at least two assays (Sequenom^™^ MassARRAY, capillary sequencing, exome or sequencing). A validated single‐nucleotide polymorphism at an incidence of 0.0008–0.02% (rs148542200) was located in the optimal position for the reverse primer: for this reason two reverse primers were used to ensure assay compatibility for all patients. A synthetic long oligonucleotide was used as a template to demonstrate that both alleles amplify equally efficiently with this PCR primer strategy (data not shown; Sigma‐Aldrich, MO).

Assays targeting hotspots in *MYOD1* (p.L122R), *H3F3A* (p.G34W), *CTNNB1* (p.T41A, p.S45F) and *GNAS1* (p.R201C, p.R201H) were also developed (Data S1).

### Digital PCR

DNA was analyzed on the QX200 Droplet Digital PCR System (Bio‐Rad, CA). 20 *μ*L reactions consisting of up to 9 *μ*L DNA, 10 *μ*L Supermix for Probes (no dUTP; Bio‐Rad), assay oligos (Tables S3–S5) and nuclease‐free water were partitioned into approximately 23,000 ~0.85 nL volume droplets and underwent 40 cycles of PCR (Table S6). Early experiments conducted as part of an optimization procedure were carried out: details are described in the supplementary material.

Between 0.16 and 6.27 mL of plasma were analyzed for each time point. If high ctDNA levels were detected in the first aliquots analyzed (≥5 mutant droplets) the remaining plasma was saved. All available plasma was analyzed if insufficient molecules were detected.

At the data acquisition stage, droplets were read with the FAM/VIC channel and RED (Rare Event Detection) setting provided by the QuantaSoft 1.7.4 software package (Bio‐Rad, CA). Droplets were manually called as mutant only, wild type only, double‐positive or template negative by the same technician throughout the study.

Positive controls and no‐template controls were included in every run. For the multiplex analyses, a single‐reaction positive control was created for all five mutations by pooling PCR amplicons from five tumor DNA samples. The mutation in each tumor was verified in a singleplex experiment. This positive control acted as an intra‐plate reference for calling droplets, as well as an inter‐plate control for intermediate precision of the assay. No‐template control results are listed in Table S7.

A commercially available pooled sample of human placental DNA was used as a mutation negative control (BioLine, London, UK) for bulk analysis of the assay background error rate. ~0.95 *μ*g of human genomic DNA was loaded across 19 reactions for total analysis of approximately 289,000 haploid copies. In total 16 mutant droplets were detected (2 “mutant only”, and 14 double positive).

Based on this background error rate (1 mutant droplet per ~18,000 wild type droplets), and the average number of molecules we assessed per time point (*n* = 1,493), blood samples were deemed to be ctDNA mutant‐positive if they had a minimum of 2 “mutant only” droplets. Samples with just a single mutant droplet were classified as equivocal and treated as negative for analysis. Analysis of cell‐free DNA from 12 donors with lipoma did not detect a single *IDH1* mutant molecule demonstrating the specificity of this assay. Mutant levels are reported in terms of mutant copies per mL of blood plasma analyzed. In order to calculate this, the variable dead volume of the BioRad platform has been factored in.

### Data analysis

Concentration values and raw droplet counts as produced by QuantaSoft were taken forward for analysis. Individual reactions with 2D plots indicative of typical assay failure or with mutant‐only droplets less than or equal to the number in the no template control combined were excluded from analysis (Fig. S1).

## Results

**Figure 1:**
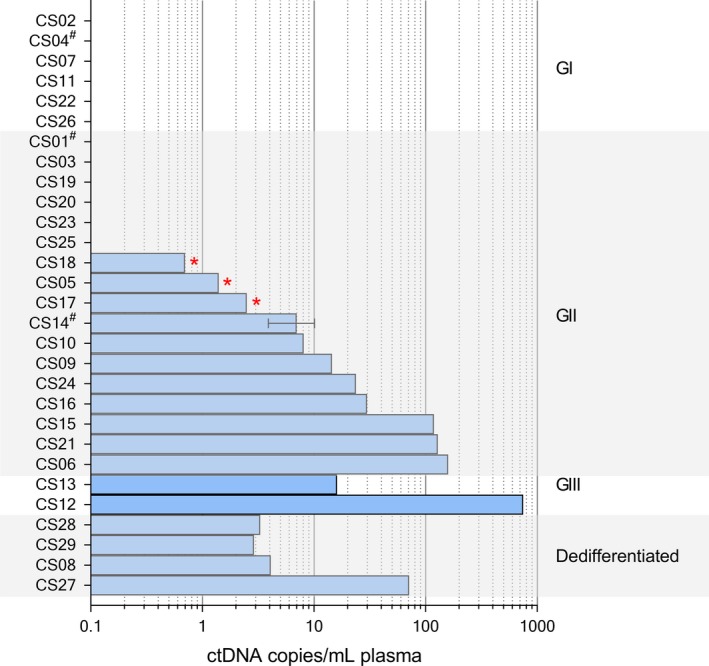
Number of mutant molecules per mL/plasma pretreatment. Patients were sorted by grade and ctDNA level. Three patients (CS01, CS04 & CS14) had multiple pretreatment timepoints. The mean of these values was taken, with 1 standard deviation displayed as error bars. Equivocal results, where only 1 mutant droplet was detected are denoted with a*. Patients with Ollier disease are identified with a^#^. CS14 had 3 separate tumors analyzed and has been grouped according to the highest grade.

Of the 29 patients with *IDH1*‐mutant CS selected for this study, 15 patients had only preoperative samples analyzed whereas a variable number of pre‐ and post‐operative serial blood samples was available throughout the course of treatment from the remaining 14 patients. In total 73 separate time points were analyzed. Preoperative ctDNA levels were detected in 14 patients including all patients with GIII (*n* = 2) and dedifferentiated (*n* = 4) CS, 8/17 patients with CS GII, but 0/6 patients with CS GI (Fig. 1).

**Figure 2:**
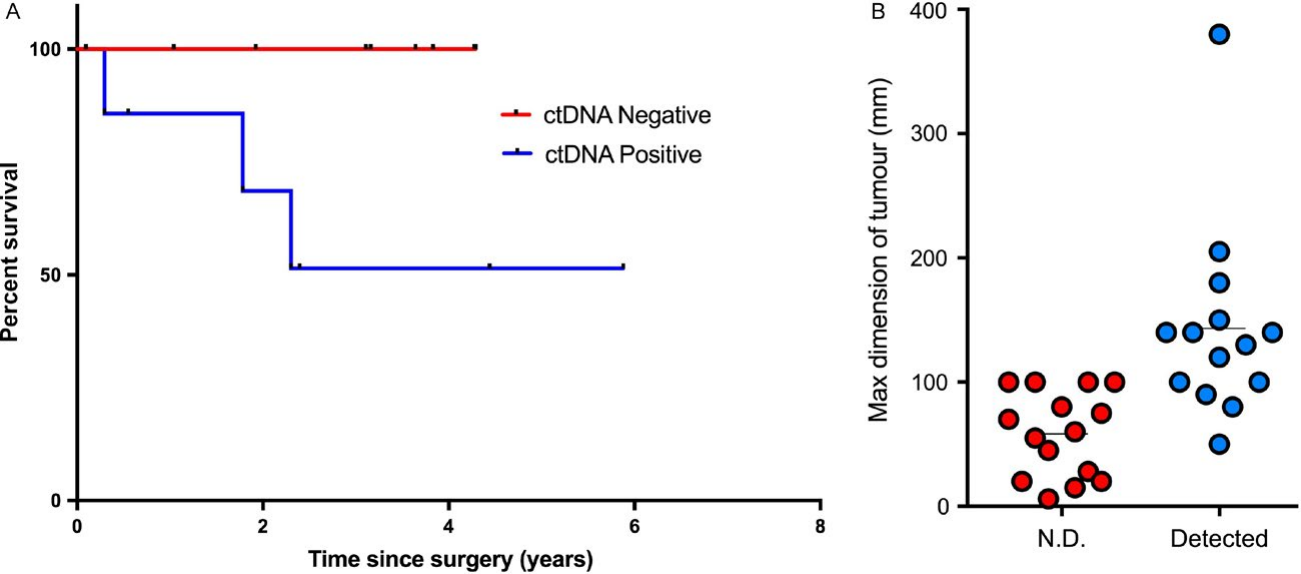
Comparison of pretreatment ctDNA levels with clinical records. (A) Kaplan–Meier analysis of patients with GII tumors with either detectable or undetectable ctDNA pretreatment, (B) maximum dimension of tumors compared with whether ctDNA could and could not be detected pretreatment. (N.D., not detected)

Correlation of the ctDNA data with clinical records revealed that detection of preoperative ctDNA in 8/17 patients with GII CS was associated with a poor prognosis compared to those in whom ctDNA was not detected: 3/8 ctDNA‐positive patients with GII disease had died by the end of the study (CS06, CS15, CS21) (*P*‐value = 0.0448 Log‐rank (Mantel‐Cox test) (Fig. 2A). A fourth patient (CS09) suffered a local recurrence, and a fifth patient (CS24) presented with metastatic disease 2 years following the primary surgery, which was subsequently excised: this patient has lung metastases. In contrast 8/9 of those in whom preoperative ctDNA was not detected were disease‐free aside from one patient whose tumor recurred as a small lesion in the phalanx (CS03). Larger tumor size (largest tumor dimension) was also associated with detection of ctDNA in individuals with GII tumors and above (*P*‐value = 0.001683, Welch Two Sample *t*‐test) (Fig. 2B).

**Figure 3:**
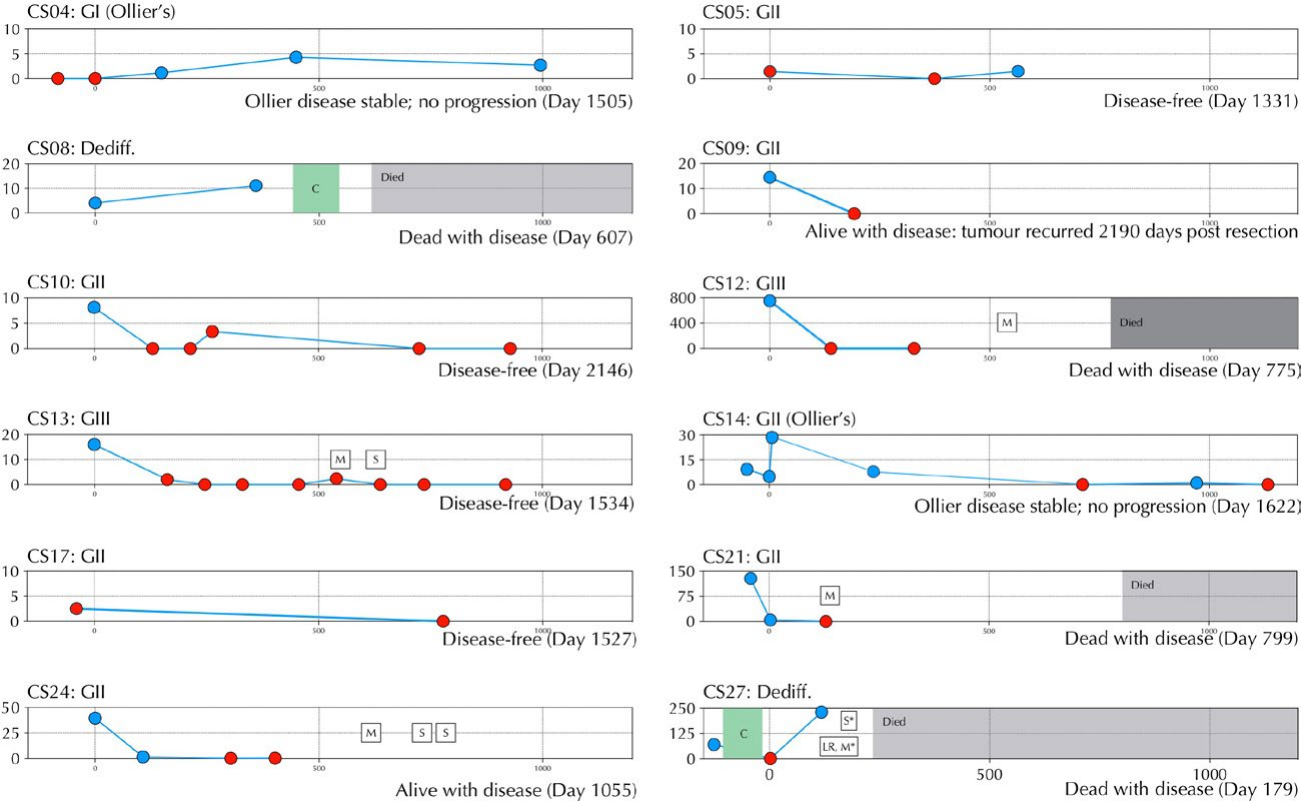
Time course figures for patients with serial analysis of ctDNA. Patient ID and grade are shown in the top left of each figure. Disease status is shown at the bottom left of each figure. *X*‐axis: days since primary surgery. *Y*‐axis: mutant IDH1 copies/mL plasma. Red points indicate a negative result of 1 or no droplets, blue points indicate ctDNA positive time points. C, chemotherapy; S, surgery, M, metastasis, LR, local recurrence. CS01 and CS20 have been excluded from this figure as all time points were negative.

Of the 14 patients whose plasma was analyzed both pre‐ and post‐surgery, ctDNA was detected presurgery in 9 (Fig. 3 and Table S1). Mutant *IDH1* ctDNA levels from 7 of these 9 patients was clearly reduced following surgery (average time when first sampled 92 days) (CS09‐192 days, CS10‐130 days, CS21‐3 days, CS24‐107 days, CS12‐139 days, CS13‐162 days & CS27‐2 days) although in patient (CS27), with a dedifferentiated CS, the ctDNA subsequently had risen significantly when measured at day 118 reflecting disease progression (local recurrence and metastatic disease) (Fig. 3). Notably there were 5 patients in whom ctDNA was detectable in their first postsurgery sample. In patient (CS14) with extensive Ollier disease the ctDNA level rose postoperatively and remained high for 236 days in the absence of clinically progressive disease, by day 712 mutant DNA was not detected and the patient remains well with stable disease 1,260 days following surgery. This patient experienced a turbulent postoperative course requiring additional surgical procedures shortly after the primary operation. Patient CS04, who also has Ollier disease, had a GI CS and *IDH1* mutant molecules detectable in plasma postoperatively on three different time points. This patient did not have detectable levels before surgery and currently has stable disease. The ctDNA levels in two patients (CS24 and CS21) with GII disease remained detectable in the first postsurgery sample (day 107 and day 3, respectively) and then became equivocal. Both these patients subsequently developed metastatic disease (510 days and 150 day after surgery, respectively). The fifth patient (CS08), who had an intralesional excision of a dedifferentiated CS was found to have significantly raised ctDNA levels when first tested postsurgery at 359 days at which time the patient had metastatic disease.

Finally, we analyzed plasma samples from 42 patients with a variety of bone and soft tissue tumors harboring a characteristic mutation of their disease using a range of hotspot digital PCR assays (Table S2). Presurgery mutant *MYOD1* p.L122R ctDNA was detected in 2/3 patients with a spindle cell rhabdomyosarcoma, all of which harbored the characteristic recurrent mutation in their tumors. The highly recurrent *H3F3A* p.G34W reported in giant cell tumors of bone was detected in ctDNA from 7/24 patients with giant cell tumors of bone all of which had previously been shown to harbor a *H3F3*A alteration. However, the ctDNA levels were typically very low (2 or 3 mutant droplets) except in one patient who had suffered a pathological fracture through the tumor. The characteristic recurrent mutations in *CTNNB1* (p.T41A, p. S45F) detected in desmoid‐type fibromatosis from five patients was not detected in plasma, nor were the recurrent *GNAS (*p.R201H, p.R201C) mutations detected in four and six patients with fibrous dysplasia and intramuscular myxoma, respectively, whose tumors harbored the relevant *GNAS* mutations.

## Discussion

We have assessed the ability to detect and quantify circulating mutant *IDH1* using a multiplexed digital PCR assay against all common *IDH1* mutations in 29 patients with CS whose tumors were known to harbor an *IDH1* mutation. We report here that there is complete correlation between the detection of presurgery ctDNA and the diagnosis of GIII and dedifferentiated CS. There is also 100% correlation between failure to detect the mutant molecules in the circulation of patients with low grade CS harboring an *IDH1* substitution. The detection of mutant *IDH1* ctDNA in patients with GII disease was much more variable, being present in 8/17 of presurgical samples from these patients. The detection of preoperative ctDNA in patients with CS GII was associated with poorer prognosis (*P*‐value = 0.0448 Log‐rank (Mantel‐Cox test) (Fig. 2A) with 5/8 patients suffering relapse of persistent disease. Overall, our findings reflect the reported behavior of CS with these grades: patients with GIII and dedifferentiated CS have a uniformly poor prognosis whereas patients with low‐grade cartilaginous tumors have an excellent outcome. In contrast, GII CS has the most unpredictable behavior and 5 year survival 1. Despite histological grading being recognized to be subject to inter‐ and intra‐observer variability 2, it is currently the best indicator of prognosis. It is therefore interesting to speculate that detection of preoperative ctDNA could be a more accurate and reproducible prognostic indicator than assessment of histological grade.

ctDNA has a half life of approximately one hour, hence detection of mutant molecules more than a few days postsurgery (depending on initial levels) indicates that they must have originated from tumor cells that have not been successfully removed. This is consistent with the evidence in some cancer types that postsurgical detection of ctDNA is more likely to be associated with disease relapse 23, 24. It is therefore noteworthy that the five patients (CS05, CS10, CS13, CS17, and CS20) who were disease‐free at the end of the study had no detectable ctDNA postsurgery (except in CS05 at a single point where 1.5 mutant molecules per mL of plasma were detected), whereas ctDNA was detectable postsurgery in the 4 patients (CS08, CS21, CS24, CS27) who either had relapsed and had persistent disease, or died of related disease. One patient (CS09) had no detectable ctDNA postsurgery but later developed a local recurrence, however, this was 6 years after the primary surgery and 66 months after the last ctDNA test. A second patient (CS12), who also had no detectable ctDNA postsurgery died with pulmonary metastases 447 days after the last ctDNA test. Furthermore the findings suggest that this assay may be useful for detecting residual and metastatic CS earlier/more sensitively than imaging, and thereby could allow earlier surgical intervention. From a therapeutic perspective, a number of new approaches are being explored including *IDH1*‐targeted therapy 25, 26. Hence, utilization of the assay may not only enable improved stratification for recruitment into trials but also the monitoring of treatment response.

Ollier disease is brought about by an early postzygotic mutation in *IDH1* or *IDH2* 14. ctDNA increased postsurgery in 2/3 Ollier patients (CS04 and CS14). However, it is not clear if the *IDH1* mutant ctDNA molecules derived from tumor or nontumor mosaic tissues. It is conceivable that the trauma of surgery resulted in release of molecules from non‐lesional *IDH1* mutant tissue, a suggestion supported by the reduction to undetectable levels in one patient who was followed for 37 months, and the finding that *IDH1* molecules were detected postsurgery in the patient CS04 with GI Ollier disease when it was not detected prior to treatment. Monitoring ctDNA levels in multiple patients with Ollier disease over a longer period and correlation with clinical progression is required to establish the value of this test in detection of disease progression.

Our finding that mutant *IDH1* ctDNA was detected in the vast majority of patients with high grade CS harboring an *IDH1* mutation indicates that preoperative detection of ctDNA could potentially replace the need for a CT‐guided needle biopsy—the conventional means of obtaining a bone tumor diagnosis. However, introducing such a change in practice could only be pursued if imaging, a vital component of the diagnostic process, correlated with ctDNA levels, and the results were confirmed in a larger cohort. The failure to detect ctDNA even if the imaging supported a diagnosis of cartilaginous tumor could be explained on the basis that ctDNA levels are not expected to be detected in GI CS and some GII CS harboring the mutation, in addition to which only 55% of conventional central CS harbor an *IDH1* mutation.

On extending our analysis to other bone and soft tissue tumors we have shown that there is a clear trend for higher levels of ctDNA in patients with higher‐grade disease. Specifically ctDNA was detected in 2/3 patients with spindle cell rhabdomyosarcoma, whereas only small numbers (2 or 3) of mutant molecules were detected in ~30% of patients with GCT of bone, a locally aggressive bone tumor. The patient with GCT of bone who had a high mutant burden had suffered a pathological fracture. Furthermore, mutant molecules were not detected in plasma of patients with desmoid‐type fibromatosis, fibrous dysplasia and intramuscular myxoma, all of which are slow‐growing indolent neoplasms which do not metastasize and in which necrosis is not found.

A limitation of this study is that *IDH1* mutants are only detected in approximately 50% of CS. Through exome sequencing of these tumors we have shown that a number of other mutations occur recurrently in a relatively small number of genes in the presence and absence of the *IDH* substitutions. These include *COL2A1* aberrations in 37% of cases, *TP53* mutations in 20% and mutations in the RB1 pathway (primarily *CDKN2A* deletion) and hedgehog signaling pathway at 33% and 18%, respectively 27. However, these genetic alterations involve numerous loci in the genes and therefore require the development of more complex assays. Although being pursued, optimization of such assays are best undertaken in cancers more common than CS 7, 8. A second limitation of the study is the variable amount of plasma DNA collected (0.2–6.3 mL) and the inconsistent time points at which this collection occurred. Future project design should include a minimum of 5 mL of plasma collected at predetermined occasions.

The results from this pilot study involving rare connective tissue tumors with highly recurrent hotspot mutations provide evidence that ctDNA may have useful applications including a more accurate prognostic indicator than is currently available, and the ability to detect residual disease/relapse postsurgery. In the latter scenario, utilisation of blood preservation tubes such as Streck or PAXgene Blood ccfDNA tubes would permit a delay in blood processing without causing a notable increase in the fraction of nontumor genomic DNA from lysed white blood cells 28. This enhances the potential for closer monitoring of patients, as blood can be drawn at their general practitioner's and sent to a central laboratory for processing and analysis. The findings argue generally for systematic collection of ctDNA from patients, particularly with rare diseases, as this will allow accelerated optimization of assays and the value of this technology to be assessed. The *IDH1* ctDNA data in patients with CS presented in this study argue for a large multicentre prospective study to confirm our findings.

## Conflict of Interest

None declared.

## Supporting information


**Table S1.** Full CS patient data.
**Table S2.** All non‐CS patient results.
**Table S3.** IDH1 multiplex assay details.
**Table S4.** GNAS multiplex assay details.
**Table S5.** SinglePlex assay details.
**Table S6.** Cycling conditions.
**Table S7.** No template control results.

**Data S1.** Methods.
**Figure S1.** An example of a rejected assay in which the reaction amplification failed.
**Figure S2.** *IDH1* R132 multiple assay testing in positive and negative control material. Droplets containing 1 or more mutant molecules are blue, droplets with one or more wild type molecules are green and the red droplets contain one or more of each. Empty droplets are grey. As demonstrated through analysis of tumor DNA with known mutations the assay is able to correctly detect each of the five mutations with very low background in wild type DNA. (A) p.R132G, (B) p.R132H, (C) p.R132L, (D) p.R132S, (E) p.R132C, (F) wild type. The wild type figure is a composite of 5 runs. This was needed to detect the extremely low background level.
