# Supplementary material for: Digital PCR analysis of circulating tumor DNA: a biomarker for chondrosarcoma diagnosis, prognostication, and residual disease detection
Source: Cancer Med. 2017 Aug 23;6(10):2194–202. doi: 10.1002/cam4.1146 (PMC5633548; doi:10.1002/cam4.1146)
Supplement: Supplementary file 2 — Data S1. Methods. Figure S1. An example of a rejected assay in which the reaction amplification failed. Figure S2. IDH1 R132 multiple assay testing in positive and negative control material. Droplets containing 1 or more mutant molecules are blue, droplets with one or more wild type molecules are green and the red droplets contain one or more of each. Empty droplets are grey. As demonstrated through analysis of tumor DNA with known mutations the assay is able to correctly detect each of the five mutations with very low background in wild type DNA. (A) p.R132G, (B) p.R132H, (C) p.R132L, (D) p.R132S, (E) p.R132C, (F) wild type. The wild type figure is a composite of 5 runs. This was needed to detect the extremely low background level. [file CAM4-6-2194-s002.doc]

**Supplementary Methods and supplementary figure 1 and 2**

**Supplementary methods**

For the first pilot digital PCR reactions DNA was heat denatured before amplification. This was to overcome the initially high dead volume of the BioRad digital PCR platform. Samples were run both with and without heat denaturation confirming no significant change in error rate. Following an update to the BioRad QX200 system the dead volume significantly decreased removing the need for heat denaturation.

During dPCR analysis the majority of samples contained suitable DNA yield. A single pre-treatment plasma sample from patient CS28 taken at day 7 had <100 wild type copies of the genome analysed (85 wild type and 1 mutant). It was therefore excluded from further analysis due to limited sensitivity.

**Supplementary tables**

**Supplementary table 1. Full CS patient data**

**Supplementary table 2. All non-CS patient results**

**Supplementary table 3. IDH1 multiplex assay details**

**Supplementary table 4. GNAS multiplex assay details**

**Supplementary table 5. SinglePlex assay details**

**Supplementary table 6. Cycling conditions**

**Supplementary table 7. No template control results**

**Supplementary figures**

Supplementary figure 1. An example of a rejected assay in which the reaction amplification failed.


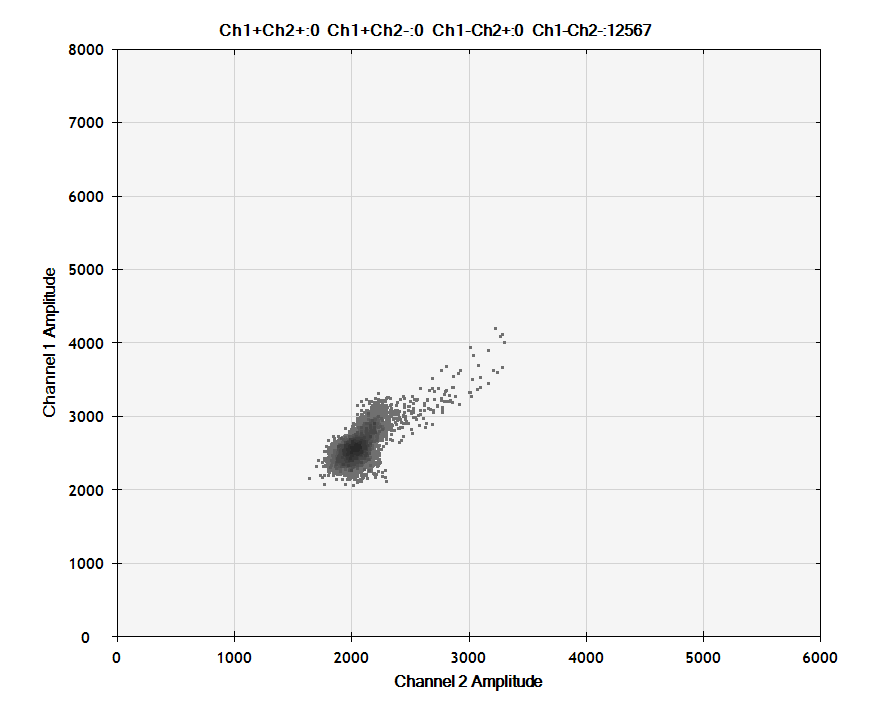


**Supplementary Figure 2.** *IDH1* R132 multiplex assay testing in positive and negative control material. Droplets containing 1 or more mutant molecules are blue, droplets with one or more wild type molecules are green and the red droplets contain one or more of each. Empty droplets are grey. As demonstrated through analysis of tumour DNA with known mutations the assay is able to correctly detect each of the 5 mutations with very low background in wild type DNA. a = p.R132G, b = p.R132H, c = p.R132L, d = p.R132S, e = p.R132C, f = wild type. The wild type figure is a composite of 5 runs. This was needed to detect the extremely low background level.


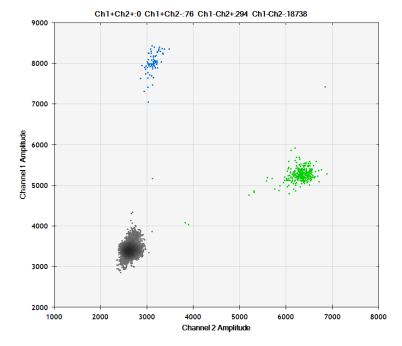

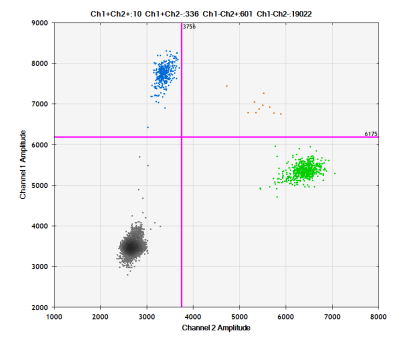

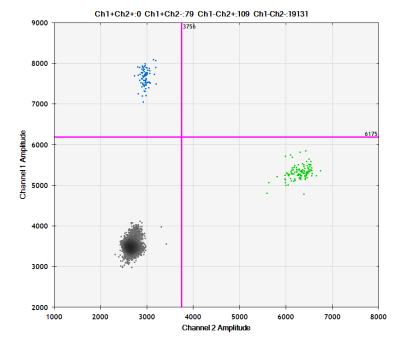

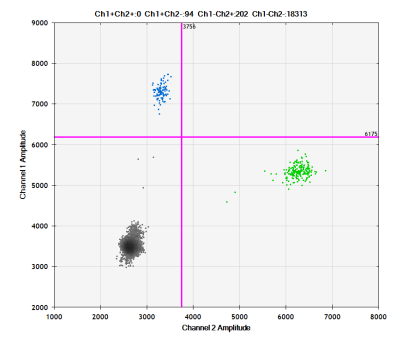

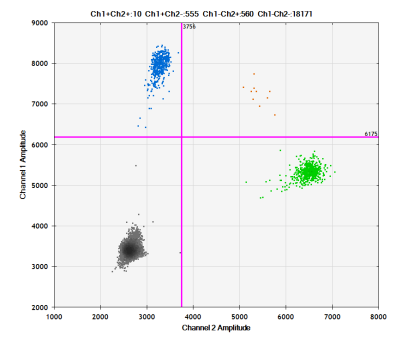

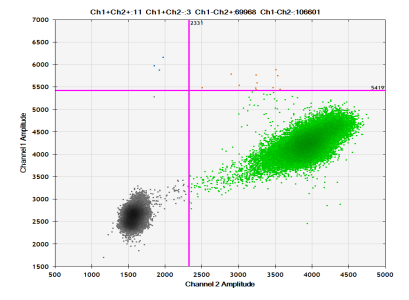


d

a

b

c

e

f
